# Hospitals Amongst the Ancient Greeks

**Published:** 1888-12-08

**Authors:** 


### Hospitals amongst the Ancient Greeks.

A few Saturdays ago Miss Jane Harrison delivered a lecture at Toynbee Hall, on "Hospitals Amongst the Greeks." Perhaps the nature of the lecture would have been better understood if Miss Harrison had said "the ancient Greeks," for her remarks were entirely devoted to the classic age. Miss Harrison began by remarking that we are accustomed to look upon the Greeks as physically perfect, given to riding on prancing horses, or posing in graceful attitudes, and would probably be indignant with her for proclaiming them as often sick and sorry. We are apt to think that there were no hospitals amongst the Greeks, and that the charity which founds such institutions is the outcome of Christianity. But hospitals existed long before the Christian era, and in Greece were closely connected with religion; the hospital was, in fact, a temple presided over by a god, and that god was Æsculapius. There is no god more misunderstood than Æsculapius; he was one of the earlier gods, and his cult was in consequence somewhat obscure; he is often therefore classed as quite a second-rate god of late origin, instead of being given the place proper to him. The reason of this was simply that his cult came to conflict with, and was ultimately subordinated to, that of Apollo. From the many images of Æsculapius which still exist, and in nearly all of which the snake is seen, there can be no doubt that he was the god of the dream-oracle of the lower world. People came to him and slept in his temple, and in their dreams he told them how to seek a cure. These temples were beautiful buildings; the ruins of one on the south side of the Acropolis have just been excavated. The precinct contained an altar for sacrifice, a long colonnade where the patients could take exercise, a shady grove, and a spring of clear water. Miss Harrison read an amusing translation from the Plutus of Aristophanes, which gave a popular account of what happened during the central ceremony of the "incubatio," or sleeping in the temple. She further pointed out how, "amid a machinery easily capable of misuse for purposes of religious fraud," there were not wanting elements of a "really experimental and progressive knowledge." First the patient had change of air and scene, and was in expectation of a cure. He would see all the stone records of past-cures erected by other grateful patients, and his mind would be cheered and alert. First he would have to bathe, then to sacrifice to the god. He would be put on a strict diet to purify himself, and would probably tell the priest who attended him all about the ailments he was suffering from. Then the patient would sleep one night within the temple, under the statue of the god. What wonder if he dreamt either of the god or of his own illness? And the priests were skilled interpreters of the slightest dreams. Some of the practical remedies prescribed by the god were excellent: Julian with hæmorrhage is to live on fir cones and honey; another dreamer with stitch in the side is to have baths and alcoholic friction, for massage was the fashion in those days as in these. There is no doubt that in the time of the Antonines, the Greek hospitals were filled with hypochondriacal men and women, even as are our modern hydropathic establishments, but in the more robust age of Plato, disease was regarded as almost synonymous with sin. To-day there are many who are sshamed of being diseased in body, but rather proud of being diseased in mind. As usual the audience was large and enthusiastic, and accorded to Miss Harrison a hearty vote of thanks. The lecture was illustrated by a magic lantern, and Miss Harrison's pleasant elocution, and skill in making popular a difficult subject, were rewarded by warm applause.

